# Stratifying nursing home care to further explore a locally validated risk factor for community-acquired pneumonia (SNAP-CAP Study)

**DOI:** 10.1017/ash.2023.466

**Published:** 2023-10-27

**Authors:** Dennis Kutsar, Nirvana Awad, Rupangi Rastogi

**Affiliations:** 1 Pharmacy, Montefiore Nyack Hospital, Nyack, NY, USA; 2 Pharmacy, Population Health Mount Sinai Health Partners, New York, NY, USA; 3 Pharmacy, Montefiore Nyack Hospital, Nyack, NY, USA

## Abstract

We observed that patients admitted to our hospital from a prestratified group of congregated living facilities had a numerically higher percentage of study-defined resistant organisms in sputum cultures compared to facilities without these characteristics [89.4% vs 78.3%, odds ratio [OR] = 2.34 (95% CI, 0.73–7.58)].

## Introduction

Admission from a nursing home was considered a risk factor for resistant organism (RO) pneumonia, under healthcare-associated pneumonia (HCAP).^1^ However, updates in 2016 removed HCAP altogether with the recommendations for institutions to identify their own locally validated risk factors for RO pneumonia, or organisms not covered by the empiric antibiotic regimen typically used to treat community-acquired pneumonia (CAP).^2^ Thus, our institution conducted an internal retrospective study, in which we identified 2 significant risk factors for RO pneumonia, which included patients who were admitted to the hospital and received antibiotics within the last 90 days, as well as those admitted from nursing homes (Supplemental Table 1). Following the results of our study, the 2019 CAP guidelines did not consider admission from nursing homes to be a significant risk factor for RO pneumonia, prompting us to re-evaluate how to approach empirically treating these patients.^3^

The CDC defines nursing homes and assisted living facilities as those able to provide medical and personal care to people unable to live independently.^4^ With varying degrees of services, it is difficult to encompass all patients coming from such facilities as equally susceptible to ROs. Therefore, we chose to categorize all facilities under the umbrella of congregated living facilities (CLFs).

We hypothesized that patients presenting from facilities with environmental characteristics similar to that of a hospital placed patients at greater risk for ROs compared to facilities that provide an environment similar to an individual’s home. Dependent care facilities met 1 or more of the following criteria: a ventilator unit, on-site dialysis, or providing inpatient rehabilitation care services (minimum rehabilitation care of 3 h per day, 5 d a week). All other facilities were defined as independent care facilities. Using these definitions, the purpose of this study was to stratify patients presenting from CLFs into the aforementioned 2 groups and to determine if admission for CAP from a dependent care facility increased the risk of ROs.

## Methods

This observational study compared the risk of ROs in patients admitted for CAP to a 251-bed community hospital in Rockland County, New York, USA. Patient records were consecutively identified from October 2015 to October 2021, with International Statistical Classification of Diseases, 10th revision codes. A list of all local CLFs was stratified into 2 groups, dependent and independent care. Patients included met objective criteria for CAP (x-ray or computed tomography of the chest performed within 48 h of admission, suggestive of infiltrates, consolidation, or opacification) and a positive sputum culture collected within 48 hours of admission. Exclusion criteria were age <18 years or fungal isolate(s).

### Study outcomes and statistical analysis

The primary outcome assessed was RO isolation from a sputum culture. Study-defined ROs were: *P. aeruginosa*, MRSA, extended-spectrum β-lactamase (ESBL) producing organisms, *Acinetobacter* species, vancomycin-resistant *Enterococcus* species, carbapenem-intermediate or resistant *Enterobacteriaceae* species (CRE), and *Stenotrophomonas maltophilia*. ROs were included regardless of susceptibilities. A secondary outcome evaluated was the type of RO isolated. Statistical calculations were performed with Vaserstats.^5^ Chi-square or Fisher’s exact probability tests were used to calculate the odds ratios and 95% confidence intervals as appropriate. A target number of 194 patients would provide 80% power to detect a difference at a 2-sided alpha level of 0.05.

## Results

Nearly 2,000 medical records were evaluated and 127 patients met eligibility criteria. There were no significant differences found in the median age of patients or in locally validated risk factors for ROs between the 2 groups (Table 1). Patients were admitted from 13 different CLFs (Supplemental Table 2). A numerically higher percentage of patients were found to have an RO isolated in the sputum when presenting from a dependent care facility compared to an independent care facility [89.4% vs 78.3%, odds ratio [OR] = 2.34 (95% CI, 0.73–7.58)]. The secondary outcome is summarized in Table 2. The most frequently isolated RO was *P. aeruginosa* (42.5%), followed by *A. baumannii* (14.9%) and *MRSA* (10.6%).

**Table 1.** Patient demographics

	Dependent care facility (*n* = 104)	Independent care facility (*n* = 23)	*P* value
Median age (range)	68 (23–98)	68 (45–95)	–
Number of patients with other locally validated risk factors* (%)	54 (51.9)	13 (56.5)	0.12

Note.

* Locally validated risk factors include either recent (≤90 d) hospitalization or systemic antibiotic use.

**Table 2.** The frequency and percentage of all ROs isolated

RO	All facilities* frequency (%)	Dependent care facility	Independent care facility
*P. aeruginosa*	60 (42.5)	55	5
*A. baumannii*	21 (14.9)	21	0
MRSA	15 (10.6)	11	4
ESBL *Proteus* spp.	13 (9.2)	12	1
ESBL *Klebsiella* spp.	11 (7.8)	9	2
ESBL *E. coli*	8 (5.7)	2	6
CRE	8 (5.7)	7	1
*S. maltophilia*	5 (3.5)	5	0

Note.

* 141 total ROs isolated in sputum cultures from all patients presenting from any facility group.

## Discussion

Admission from a dependent care facility did not significantly increase the risk for ROs compared to admission from an independent care facility. To our knowledge, this is the first study that objectively stratified CLFs in order to better study the differences in organism prevalence. The criteria used to define a dependent care facility (on-site ventilator unit, dialysis and/or providing acute rehabilitation) were based on evidence demonstrating increased risk of ROs. A recent study showed that the acquisition of ROs is common among patients in an outpatient dialysis unit (occurring in 40% of patients over a 6-m period).^6^ Through molecular analysis, similar strains of ROs between patients and the environment were identified, supporting a strong likelihood of cross-transmission within dialysis facilities. Lin and colleagues investigated the prevalence of carbapenemase-producing organisms (CPO) in ventilator skilled nursing facilities (vSNFs).^7^ Across 7 vSNFs, the overall prevalence of CPO carriage was found to be 27%, significantly higher in ventilator wards (40%) compared to skilled wards (10%), *P* < 0.001. The investigators concluded that individuals residing in SNFs face increased susceptibility to CPO carriage, with the greatest vulnerability for those residing in a ventilator ward. In regards to the acute rehabilitation services criteria, a study conducted by Gontjes and colleagues investigated the presence and transmission of multidrug-resistant organisms (MDROs) within CLFs and quantified the organism burden found in the common areas and on the gym equipment.^8^ Of the 60 rehabilitation gym sampling visits, it was observed that 33 (55%) had at least one MDRO-positive specimen. This study demonstrated that rehabilitation gyms can be an MDRO reservoir with significant microorganism transfer potential (transfer occurred in 17.1% of opportunities).

At our hospital, we identified that patients admitted from CLFs were at high risk for RO pneumonia. A majority of these patients were from dependent care facilities (82%), which may be a reason that this risk factor was identified. Limitations of this study include a small sample size, which was likely associated with the difficulty in collecting high-quality sputum cultures,^9^ and the reliance on patient medical records for information. An ideal analysis would have excluded patients with known risk factors for ROs (recent hospitalization and recent antibiotic use), however, this is less reflective of the real world and would greatly limit sample size. Future research should consider the history of colonization or infection with a RO as well. While we aimed to control for relevant demographic variables, the absence of comorbidity information remains a limitation that could limit the generalizability of our findings. Our study did not meet power to detect a significant difference in the primary outcome, but we believe that stratification of facilities based on environmental characteristics, medical services, or other criteria may be an important step in identifying prehospital settings associated with higher risk for ROs. The stratifications presented in this study are preliminary and require further validation prior to implementation into clinical practice. After evaluating this data, we encourage other institutions to review microbial cultures from local CLFs so that facilities with higher percentages of ROs may be identified and characteristics of these facilities may be reported in order to identify trends in antimicrobial resistance. The information will allow for better guidance of empiric antibiotic selection for patients admitted from more precisely stratified CLFs.

## Supporting information

Kutsar et al. supplementary material Kutsar et al. supplementary material

## Supplementary material

For supplementary material accompanying this paper visit http://doi.org/10.1017/ash.2023.466. click here to view supplementary material

## References

[1] American Thoracic Society; Infectious Diseases Society of America. Guidelines for the management of adults with hospital-acquired, ventilator-associated, and healthcare-associated pneumonia. Am J Respir Crit Care Med 2005;171:388–416. 15699079 10.1164/rccm.200405-644ST

[2] Kalil AC , Metersky ML , Klompas M , et al. Management of adults with hospital-acquired and ventilator-associated pneumonia: 2016 clinical practice guidelines by the Infectious Diseases Society of America and the American Thoracic Society. Clin Infect Dis 2016;63:e61–e111 27418577 10.1093/cid/ciw353 PMC4981759

[3] Metlay JP , Waterer GW , Long AC , et al. Diagnosis and treatment of adults with community-acquired pneumonia. An official clinical practice guideline of the American Thoracic Society and Infectious Diseases Society of America. Am J Respir Crit Care Med 2019;200:e45–e67. 31573350 10.1164/rccm.201908-1581ST PMC6812437

[4] CDC Nursing Homes. Centers for disease control and prevention website. https://www.cdc.gov/longtermcare/index.html. Published 2020. Accessed August 1, 2023.

[5] Lowry R. VassarStats: website for statistical computing. http://www.vassarstats.net. Published 2022. Accessed April 4, 2022.

[6] D’Agata EMC. Addressing the problem of multidrug-resistant organisms in dialysis. Clin J Am Soc Nephrol 2018;13:666–668. 29567862 10.2215/CJN.13781217 PMC5968912

[7] Lin MY , Froilan MC , Lolans K , et al. the importance of ventilator skilled nursing facilities (vSNFs) in the regional epidemiology of carbapenemase-producing organisms (CPOs). Open Forum Infect Dis 2017;4:S137–S138.

[8] Gontjes KJ , Gibson KE , Lansing B , Cassone M , Mody L. Contamination of common area and rehabilitation gym environment with multidrug-resistant organisms. J Am Geriatr Soc 2020;68:478–485. 31851386 10.1111/jgs.16284 PMC9190293

[9] García-Vázquez E , Marcos MA , Mensa J , et al. Assessment of the usefulness of sputum culture for diagnosis of community-acquired pneumonia using the PORT predictive scoring system. Arch Intern Med 2004;164:1807–1811. 15364677 10.1001/archinte.164.16.1807

