# Supplementary material for: Stratifying nursing home care to further explore a locally validated risk factor for community-acquired pneumonia (SNAP-CAP Study)
Source: Antimicrob Steward Healthc Epidemiol. 2023 Oct 27;3(1):e191. doi: 10.1017/ash.2023.466 (PMC10654936; doi:10.1017/ash.2023.466)
Supplement: Kutsar et al. supplementary material [file S2732494X23004667sup001.pdf]

**Supplementary Table 1. Unpublished Results of an Internal Study Conducted to Identify Locally Validated Risk Factors**		
Risk Factor Assessed	Odds Ratio for RO (95% CI)	*p* value
Admission From a Congregated Living Facility	10.2 (3.82 – 27.21)	<0.0001
Antibiotics within 90 Days	4.02 (1.5 – 10.6)	0.005
Previous hospitalization ≥ 2 days in the past 90 days	3.82 (1.56 – 9.36)	0.004
Chronic dialysis	1.67 (0.37 – 7.45)	0.71
Immunosuppression	1.9 (0.58 – 6.21)	0.38
ICU admission within 24 hours	2.52 (0.99 – 6.37)	0.07

**Supplementary Table 2. Distribution of Patients By Facility Type**	
Facility Characteristics	Number of Patients
On-site dialysis or ventilator unit	97
Subacute rehabilitation (<3 hours/day rehab care)	18
Inpatient rehabilitation facility (>3 hours/day rehab care)	7
Assisted living facility	4
Inpatient psychiatric facility	1
